# Euglycemic Diabetic Ketoacidosis in Pregnancy

**DOI:** 10.5811/cpcem.2019.9.43624

**Published:** 2019-11-15

**Authors:** Júlio César Garcia de Alencar, Geovane Wiebelling da Silva, Sabrina Correa da Costa Ribeiro, Júlio Flavio Meirelles Marchini, Rodrigo Antonio Brandao Neto, Heraldo Possolo de Souza

**Affiliations:** São Paulo University, Department of Emergency Medicine, São Paulo, Brazil

## Abstract

The clinical presentation of diabetic ketoacidosis in pregnancy (DKP) is similar to that observed in nonpregnant women, although reports suggest the presenting blood glucose level may not be as high. It is hypothesized that lower, maternal fasting glucose levels are a result of both the fetus and the placenta consuming glucose. We report the case of a 38-year-old woman gravida 2, para 0, abortion 1 with type 1 diabetes who had euglycemic diabetic ketoacidosis and review the literature on DKP, with a focus on diagnosis, treatment, and monitoring of the mother and fetus.

## INTRODUCTION

Diabetic ketoacidosis in pregnancy (DKP) is a serious complication that develops because of relative or absolute insulin deficiency and a simultaneous increase in counter-regulatory hormones.^1^ DKP is usually observed in patients with type 1 diabetes, but it can also occur in those with type 2 and gestational diabetes.^2^ It is likely to be precipitated by specific factors, such as hyperemesis gravidarum, starvation, infections, nonadherence to insulin therapy, and certain medications (e.g., steroids).^2^,^3^

Euglycemic DKP is a rare situation where the patient presents with diabetic ketoacidosis and normal or subnormal blood glucose levels. The putative mechanisms are a combination of glucose consumption by the fetoplacental unit, increased renal glucose losses, increased maternal usage of blood glucose, or a dilutional effect.^4^–^6^

## CASE REPORT

A 38-year-old woman (gravida 2, para 0, abortus 1) was admitted to the emergency department at 34 weeks of pregnancy with nausea and vomiting. The patient had type 1 diabetes (diagnosed at age seven) treated with insulin glargine and lispro. She had a history of nonadherence to insulin therapy and one previous intensive care unit admission due to DKP at 22 weeks of the same gestation.

The patient sought care complaining of nausea, vomiting, anorexia, and abdominal pain of three days’ duration. She had not administered any insulin in the prior seven days. She presented drowsy and dehydrated, with a stable blood pressure of 100/70 millimeters of mercury (mmHg) although tachycardic (140 beats per minute) and tachypneic (32 breaths per minute). The capillary glucose level was 82 milligrams per deciliter (mg/dL) (reference range, <200 mg/dL). Laboratory results showed pH 7.25 (reference range 7.35–7.45), bicarbonate 10 milliequivalent per liter (mEq/L) (reference range 21–27 mEq/L), base excess −14.9 (reference range −2 to +2), blood glucose 80 mg/dL, and urine strongly positive for ketones. She had no signs of infectious processes. Point-of-care abdominal ultrasound showed a single fetus in cephalic presentation with limb movements, a fetal heart rate of 160 beats per minute, and normal amniotic fluid index.

The patient was rehydrated with 5% dextrose and 0.9% sodium chloride solution administered at 500 milliliters per hour (mL/h) for the first hour, followed by 250 mL/h in the succeeding four hours, and then tapered to 125 and 75 mL/h until resolution of diabetic ketoacidosis. Hydration was titrated to ensure a urine output of 0.5 mL/kilogram (kg)/h (measured via indwelling catheter). Potassium was administered at 20 millimoles (mmol)/L/h until the fourth hour of resuscitation. A continuous intravenous (IV) infusion of regular insulin was started at 7 units (U)/h.

Clinical condition and laboratory data were monitored continuously. After 24 hours, laboratory results showed pH 7.44, bicarbonate 21 mEq/L, and blood glucose levels in the normal range. Nevertheless, fetal monitoring showed sustained bradycardia after 22 hours in the ED and the obstetrics team opted for an emergency cesarean section, which was performed uneventfully. The patient was discharged 48 hours after cesarean section with a new insulin prescription. The neonate was discharged 14 days later.

## DISCUSSION

The Joint British Diabetes Societies Inpatient Care Group requires laboratory data to confirm the diagnosis of DKP. The diagnostic criteria are as follows: 1) blood ketone level ≥3.0 mmol/L or urine ketone level >2+; 2) blood glucose level >200 mg/dL or known diabetes mellitus; and 3) bicarbonate level <15.0 mEq/L and/or venous pH< 7.3.^3^

The emergency physician must be able to recognize and treat DKP even when the patient is normoglycemic. Nausea, vomiting, and decreased caloric intake in an otherwise healthy pregnant diabetic woman should prompt evaluation to rule out ketosis.^7^ The patient should receive large-bore IV access and continuous monitoring of echocardiogram and pulse oximetry.^8^ Treatment consists of fluid replacement with isotonic saline at 10–15 mL/kg/h for the first hour,^1^ after which the rate should be adjusted according to the hemodynamic status of the patient, blood pressure, and urine output. The goals are a systolic blood pressure ≥90 mmHg and a urine output 0.5 mL/kg/h, monitored using an indwelling catheter.^3^

Management of euglycemic DKP follows the same principles as that of regular DKP.^3^ Insulin should not be delayed except for hypokalemia (see below). Therefore, in euglycemic ketoacidosis, 5% dextrose is necessary from the start of treatment.^9^ At our facility, we start a regular insulin infusion at an initial rate of 0.1 U/kg/h (to a maximum of 15 U/h).^3^ A priming bolus is not required.^3^ If metabolic targets are not achieved, the insulin infusion is increased by 1 U/h until ketones reach the desired level.^3^ Once DKP is resolved, we administer a first dose of subcutaneous rapid-acting insulin and a meal, and discontinue the insulin infusion 30–60 minutes later. 3

If the serum potassium is below 3.3 mEq/L, we start potassium chloride replacement before starting the insulin infusion.^3^ Patients with a normal urine output and serum potassium level <5.5mEq/L should receive potassium chloride in order to maintain their potassium level in the range of 4–5 mEq/L.^3^ Although whole-body phosphate is decreased, replacement is not necessary unless the serum level is <1 mg/dL or the patient develops cardiopulmonary effects of hypophosphatemia.^3^ Administration of bicarbonate is not recommended, as there is no evidence of a beneficial effect and it may be harmful to the patient and the fetus.^3^

Capillary glucose should be monitored hourly to avoid hypoglycemia. Blood ketones should be monitored hourly for the first six hours; the goal is to decrease rate ketones by at least 0.5 mmol/L every hour. Other biochemical parameters, such as pH, bicarbonate, and serum potassium, can be monitored every two hours. The goal for bicarbonate levels is an increase of 3 mEq/L every hour.^3^

**CPC-EM Capsule**

What do we already know about this clinical entity?

Diabetic ketoacidosis in pregnancy (DKP) develops because of relative or absolute insulin deficiency and a simultaneous increase in counter-regulatory hormones.

What makes this presentation of disease reportable?

We report the case and review literature regarding DKP in terms of diagnosis, treatment, and maternal and fetal monitoring.

What is the major learning point?

On suspicion of DKP, the physician must confirm the diagnosis, immediately start treatment, and continuously monitor the mother and fetus.

How might this improve emergency medicine practice?

The emergency physician should be able to recognize and treat diabetic ketoacidosis in pregnant women even if they are normoglycemic.

The fetal effects of DKP stem from a combination of severe maternal dehydration and acidosis, and may reduce uteroplacental perfusion. Maternal electrolyte imbalances may result in fetal cardiac arrhythmias.^10^ The necessary frequency of fetal monitoring is unknown, and no definite recommendations are currently available.^10^ Therefore, individualized care with a multidisciplinary approach is recommended. The decision to deliver should be individualized and based on evaluation of the maternal clinical status, laboratory results, fetal gestational age, cardiotocography, and ultrasound.^11^

## CONCLUSION

DKP is a medical emergency. The emergency physician should be able to recognize and treat it even when the patient is normoglycemic (euglycemic DKP). On suspicion of DKP, the diagnosis should be confirmed with laboratory tests. In addition to basic measures such as airway maintenance and hemodynamic stabilization, treatment consists of large-bore IV access, glucose and insulin infusion, correction of electrolytic imbalances, and continuous maternal and fetal monitoring.
